# Neurologic manifestations of nonhospitalized patients with COVID‐19 in Wuhan, China

**DOI:** 10.1002/mco2.13

**Published:** 2020-07-02

**Authors:** Hong Ding, Shanye Yin, Yahong Cheng, Yilun Cai, Weishan Huang, Wenjun Deng

**Affiliations:** ^1^ Key Laboratory of Combinatorial Biosynthesis and Drug Discovery Wuhan University School of Pharmaceutical Sciences Wuhan University Wuhan China; ^2^ Department of Medical Oncology, Dana Farber Cancer Institute Harvard Medical School Boston Massachusetts; ^3^ Medical School of Wuhan University of Technology Wuhan China; ^4^ Department of Pathobiological Sciences, School of Veterinary Medicine Louisiana State University Baton Rouge Louisiana; ^5^ Department of Microbiology & Immunology, College of Veterinary Medicine Cornell University Ithaca New York; ^6^ Department of Neurology, Massachusetts General Hospital Harvard Medical School Boston Massachusetts

Dear Editor:

The coronavirus disease 2019 (COVID‐19) caused by the highly infectious severe acute respiratory syndrome coronavirus 2 (SARS‐CoV‐2) had spread to every continent, with more than 4 million confirmed cases all over the world by May 9, 2020. The typical clinical syndromes of COVID‐19 involve viral pneumonia associated with acute respiratory distress syndrome (ARDS), which is the leading cause of morbidity and mortality at the advanced disease stage.^1^, ^2^ COVID‐19 manifests a complex disease epidemiology and pathophysiology^2^, ^3^; the viral infection not only affects the respiratory system but also causes cardiovascular injuries^1^, ^2^ and neurological manifestations,^4^ a notion with important implications for diagnosis, patient care, and prognosis.

There is, however, little documented information of disease presentations, progression, and potential sequela of COVID‐19 patients who went through self‐quarantine and recovered from the infection. To this end, confirmed COVID‐19 cases (by RT‐PCR) who were not eligible for hospitalization were recruited in this study and closely followed up for 12–14 weeks since the disease onset. The clinic information was extracted from medical records and confirmed or supplemented through a questionnaire‐based survey through social messaging app interviews to determine the detailed symptoms. Respiratory (fever, dry cough, and shortness of breath), cardiac (chest pain/tightness and palpitation), and neurologic symptoms involving central nervous system (CNS) manifestations (dizziness, headache, and impaired consciousness) and peripheral nervous system (PNS) manifestations (e.g., taste/smell/vision impairment and nerve pain) were specified using an online survey.

A total of 153 nonhospitalized patients with confirmed COVID‐19 (tested positive by RT‐PCR) voluntarily participated in this ongoing longitudinal study (mean age, 44.9 years [range, 18‐79 years]; 36.6% male). Eighty (52.3%) patients had fever at onset of illness, 77 (50.3%) dry cough, 36 (23.5%) shortness of breath, and 116 (75.8%) viral pneumonia in lung computerized tomography (CT) images, 17 (11.1%) anorexia, 42 (27.5%) diarrhea, 29 (19%) pharyngalgia, 9 (5.9%) nausea, 78 (51%) fatigue, 32 (20.9%) chest pain, 45 (29.4%) chest tightness, and 53 (34.6%) palpitation. Notably, our results revealed that neurologic manifestations were common in nonhospitalized patients in Wuhan (total, 77.8%; CNS, 46.7%; PNS, 69.3%), and the rates were higher than previously reported in hospitalized patients from the same area (36.4% had neurologic manifestations),^4^ probably as a result of our meticulous recording and long‐term following‐up revealing more details that were preciously overlooked.

Altogether we identified 96 (62.7%) patients who had both clear respiratory symptoms and lung infection by lung CT images (pneumonia cases). In contrast, the other patients (57, 37.3%) showed no/minor respiratory manifestations or lung infection and thus were defined as nonpneumonia cases. As shown in Table 1, compared to pneumonia cases, nonpneumonia cases were less likely to develop symptoms of immune system response such as fever (5.4% vs 80.2%, *P* = 5.04 × 10^−21^); however, they showed the same levels of, or even severer, symptoms associated with neurological manifestations. Specifically, similar rate of CNS manifestations (47.4% vs 45.8%, *P* = .87) but higher rate of PNS manifestations (78.9% vs 63.5%, *P* = .05). PNS symptoms including nerve pain (56.3% vs 56.1%, *P* = 1), excessive sweating (29.2% vs 22.8%, *P* = .45), and smell/taste impairment (21.1% vs 16.7%, *P* = .52) were common in both groups. In contrast, nonpneumonia cases were more likely to have tingling and numbness (22.8% vs 4.2%, *P* = 8.0 × 10^−4^) and vision impairment (8.8% vs 0%, *P* = .01) than pneumonia cases, indicating more frequent autonomic nerve damage.

**TABLE 1:** Demographics and clinical characteristics of non‐hospitalized COVID‐19 patients in Wuhan

	No. (%)			
Characteristic	Total	Pneumonia	Nonpneumonia	*P* value
No. of patients	153	96 (62.7)	57 (37.3)	N.A.
Age, mean [range], years	44.9 [18‐79]	44.2 [18‐79]	42.3 [28‐69]	1
Male	56 (36.6)	40 (41.7)	16 (28.1)	.11
Fever	80 (52.3)	77 (80.2)	3 (5.4)	5.04 × 10^−21^
Dry cough	77 (50.3)	66 (68.8)	11 (19.3)	2.37 × 10^−9^
Shortness of breath	36 (23.5)	35 (36.5)	1 (1.8)	9.78 × 10^−8^
CT findings^*^	116 (75.8)	96 (100)	20 (35.1)	2.82 × 10^−21^
Anorexia	17 (11.1)	11 (11.5)	6 (0.5)	1
Diarrhea	42 (27.5)	27 (28.1)	15 (26.3)	.85
Pharyngalgia	29 (19)	19 (19.8)	10 (17.5)	.83
Nausea	9 (5.9)	2 (2.1)	7 (12.3)	.01
Fatigue	78 (51)	51 (53.1)	27 (47.4)	.51
Chest pain	32 (20.9)	23 (24)	9 (15.8)	.3
Chest tightness	45 (29.4)	33 (34.4)	12 (21.1)	.1
Palpitation	53 (34.6)	37 (38.5)	16 (28.1)	.29
Nervous system symptoms				
Any	119 (77.8)	71 (74.0)	48 (84.2)	.16
CNS	71 (46.4)	44 (45.8)	27 (47.4)	.87
Headache	48 (31.4)	33 (34.4)	15 (26.3)	.37
Dizziness	18 (11.8)	12 (12.5)	6 (10.5)	.8
PNS	106 (69.3)	61 (63.5)	45 (78.9)	.05
Impaired taste and smell	28 (18.3)	16 (16.7)	12 (21.1)	.52
Impaired vision	5 (3.3)	0 (0)	5 (8.8)	.01
Nerve pain	86 (56.2)	54 (56.3)	32 (56.1)	1
Arthralgia	6 (3.9)	3 (3.1)	3 (5.3)	.67
Tingling and numbness	17 (11.1)	4 (4.2)	13 (22.8)	8.0 × 10^−4^
Excessive sweating	41 (26.8)	28 (29.2)	13 (22.8)	.45
Muscle weakness	10 (6.5)	4 (4.2)	6 (10.5)	.18
Disease duration^†^				
0‐1 week	9 (5.9)	5 (5.2)	4 (7)	.73
1–2 weeks	34 (22.2)	25 (26)	9 (15.8)	.16
2–3 weeks	44 (28.8)	39 (40.6)	5 (8.8)	1.56 × 10^−5^
3–4 weeks	24 (15.7)	13 (13.5)	11 (19.3)	.36
4–8 weeks	25 (16.3)	14 (14.6)	11 (19.3)	.5
>8 weeks	17 (11.1)	0 (0)	17 (29.8)	9.06 × 10^−9^
IgM/IgG serology^‡^				
No. of patients	77	49	28	N.A.
IgM (−) IgG (+)	31 (40.3)	27 (55.1)	4 (14.3)	5.91 × 10^−4^
IgM (+) IgG (+)	15 (19.5)	10 (20.4)	5 (17.9)	1
IgM (+) IgG (−)	10 (13)	2 (4.1)	8 (28.6)	.004
IgM (−) IgG (−)	21 (27.3)	10 (20.4)	11 (39.3)	.11

Abbreviations: CNS, central nervous system; PNS, peripheral nervous system.

* CT findings of viral pneumonia such as ground‐glass opacities and consolidation.

† Disease duration indicates the time from onset of the symptoms until the symptoms disappeared.

‡ IgM/IgG serological tests were performed 7–8 weeks post disease onset using colloidal gold antibody test kit.

Moreover, as shown in Table 1, nonpneumonia cases, compared to pneumonia cases, were associated with prolonged disease courses (>8 weeks, 29.8% vs 0%, *P* = 9.06 × 10^−9^) and impaired IgG seroconversion (i.e., higher IgM (+) IgG (−) [28.6% vs 4.1%, *P* = .004] and lower IgM (−) IgG (+) [14.3% vs 55.1%, *P* = 5.91 × 10^−4^]) during recovery (7‐8 weeks post disease onset), suggesting insufficient virus‐specific antibody response and potential latent infection. Disease relapse had been observed in 34 (22.2%) patients (20/96 [20.83%] pneumonia patients and 14/57 [24.56%] nonpneumonia patients), including three subjects with IgG (+), suggesting that the antibody cannot fully protect these patients from relapse/reinfection. Four of 96 (4.16%) pneumonia patients and three of 57 (5.26%) nonpneumonia patients remained/returned viral positive after 6 weeks of disease onset.

Our study revealed that a large subset of nonhospitalized COVID‐19 patients in Wuhan developed minimal respiratory symptoms but exhibited neurologic manifestations as the major symptoms. This is distinct from patients infected by SARS, for whom neurologic complications appeared quite late in established disease. Until now, the mechanisms of development and function, as well as the levels and duration of SARS‐CoV‐2‐specific immune responses remain unknown. It is also unclear whether the patients tested positive again by PCR tests had been reinfected after the initial viral clearance or undergone internal reactivation of the initial infection. Notably, patients with only neurologic manifestations showed impaired IgG seroconversion, indicating that their virus‐eliminating immune response may not work that well. One possible explanation is that SARS‐CoV‐2 might function as a neurotropic virus and establish prolonged latent infections in the neurons, similar to that observed in herpes zoster caused herpes simplex virus.^5^ Whether these patients can reactivate viral shedding and become capable of spreading the virus again, after their symptoms resolved and relapsed, still awaits further investigation. Nevertheless, based on our observations of a number of relapsed patients, none of their close contacts had contracted the infection via interactions with these patients.

Of special note, due to lack of awareness of the atypical neurologic symptoms, COVID‐19 patients could be diagnosed and treated improperly. For example, antidepressants have been prescribed for a number of patients included in this study, trying to relieve their long‐lasting neurological symptoms. Moreover, the current public education of COVID‐19 patient screening emphasizes respiratory symptoms; therefore, the number of suspected COVID‐19 patients who did not go to hospital for testing could be substantial because they do not have any respiratory symptoms, even if they have neurologic manifestations. Furthermore, the standard operation procedure (SOP) in most hospitals uses PCR tests with pharyngeal swabbing samples to diagnose COVID‐19, while patients who have no typical upper respiratory symptoms but suffer neurologic manifestations might be tested negative by following this SOP. These patients represent important hidden sources of contagion. Failure to identify and accurately manage patients who had no/light respiratory symptoms, but clear neurologic manifestations could lead to prolonged pandemic and even worse, pandemic resurge. For pneumonia patients, it is not clear whether respiratory symptoms or neurologic symptoms came first, as these patients tended to focus on the respiratory symptoms and might have neglected the milder neurologic manifestation during the initial stage of disease onset. Nevertheless, it is clear that that some patients had neurologic manifestations as the first symptom.

The limitations of this study include the small number of patients and a putative patient selection bias. It is possible that asymptomatic patients who really had no symptoms were less likely to participate our survey, so the rates of different manifestations could be higher than in the overall COVID‐19 patients. Moreover, it is less likely for elderly people who do not often use the social messaging app to participate in our study. Nevertheless, our findings ring the danger alarm that neurologic manifestations should be taken into account for COVID‐19 clinical diagnosis, patient care, and following up self‐quarantined and discharged patients.

## CONFLICT OF INTERESTS

W.H. receives research support from MegaRobo Technologies Corporation. The other authors claim no conflict of interest.

## ETHICAL APPROVAL

The study was approved by Wuhan University School of Pharmaceutical Sciences Ethics Committee.

## AUTHOR CONTRIBUTIONS

Hong Ding, Shanye Yin, Weishan Huang, and Wenjun Deng developed the study concept and drafted the paper. Hong Ding, Yahong Cheng, and Shanye Yin collected the epidemiological and clinical data. Shanye Yin and Yahong Cheng summarized and analyzed all the data..
